# Supplementary material for: Geometric effects in gas vesicle buckling under ultrasound
Source: Biophys J. 2022 Sep 7;121(21):4221–8. doi: 10.1016/j.bpj.2022.09.004 (PMC9674984; doi:10.1016/j.bpj.2022.09.004)
Supplement: Document S1. Figures S1–S8 [file mmc1.pdf]

Biophysical Journal, Volume *121*

Supplemental information

Geometric effects in gas vesicle buckling under ultrasound

Hossein Salahshoor, Yuxing Yao, Przemysław Dutka, Nivin N. Nyström, Zhiyang Jin, Ellen Min, Dina Malounda, Grant J. Jensen, Michael Ortiz, and Mikhail G. Shapiro

Geometric effects in gas vesicle buckling under ultrasound

Hossein Salahshoor*[1], Yuxing Yao*[2], Przemysław Dutka*[2,3], Nivin N. Nyström[2], Zhiyang Jin[2], Ellen Min[2], Dina Malounda[2], Grant J. Jensen[3,4], Michael Ortiz[1], Mikhail G. Shapiro[2,5]**

[1]Division of Engineering and Applied Science, California Institute of Technology, Pasadena, CA 91125, USA
[2]Division of Chemistry and Chemical Engineering, California Institute of Technology, Pasadena, CA 91125, USA
[3]Division of Biology and Biological Engineering, California Institute of Technology, Pasadena, CA 91125, USA
[4]College of Physical and Mathematical Sciences, Brigham Young University, Provo, UT 84602, USA
[5]Howard Hughes Medical Institute, Pasadena, CA 91125, USA
*Equal contribution
**Correspondence should be addressed to M.G.S. (mikhail@caltech.edu)

Supporting material

a

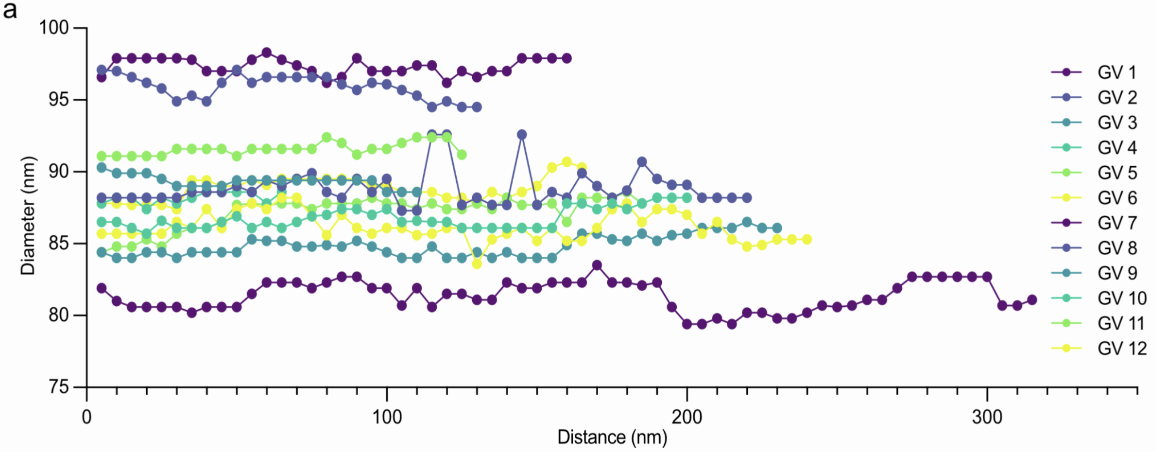

b

ID	Average diameter (nm)	Standard deviation (nm)	SD as diameter fraction (%)
GV 1	81.4	1.0	1.3
GV 2	95.9	0.8	0.9
GV 3	89.3	0.4	0.5
GV 4	86.8	0.7	0.8
GV 5	91.6	0.4	0.5
GV 6	86.2	1.0	1.1
GV 7	97.3	0.6	0.6
GV 8	88.9	1.2	1.4
GV 9	84.9	0.7	0.9
GV 10	88.2	0.4	0.5
GV 11	87.2	1.1	1.3
GV 12	88.9	0.8	0.9
		Average (%)	0.8

Figure S1. Diameter consistency analysis. (a) Diameter measurements of individual GVs. For each individual GV, the diameter was measured at increments of 10 nm along the main axis. (b) Table of summarized data showing diameter consistency across individual GVs.

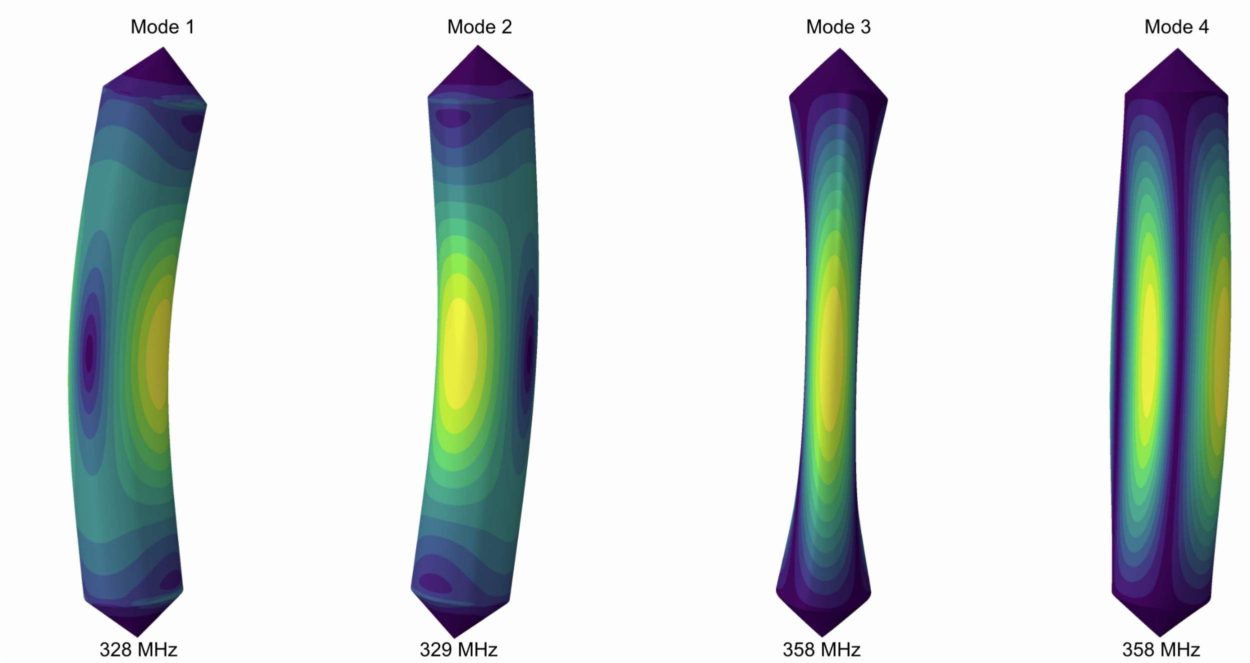

Figure S2. Resonant frequencies of gas vesicle with length and diameter of 500 nm and 85 nm, respectively.

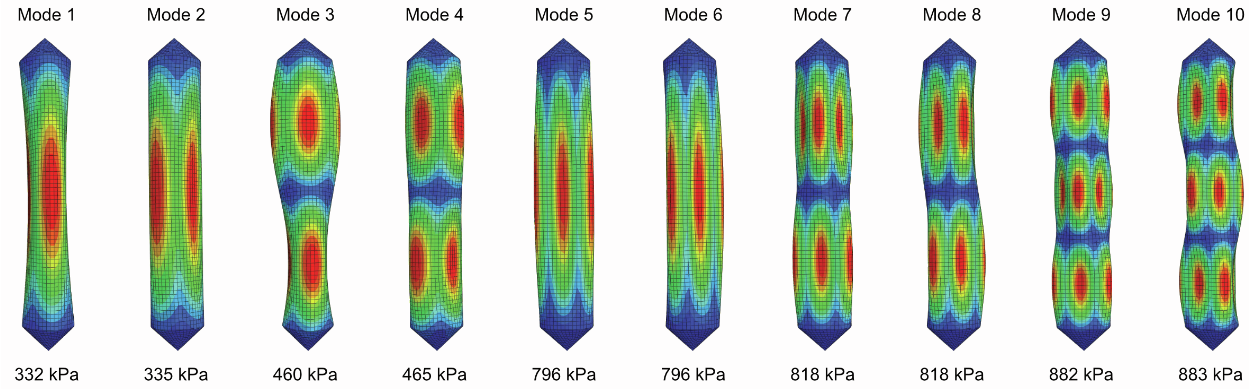

Figure S3. The first ten modes of buckling (*i.e.*, eigenvectors) and the corresponding threshold buckling pressures (*i.e.*, eigenvalues) obtained through linear buckling analysis (LBA).

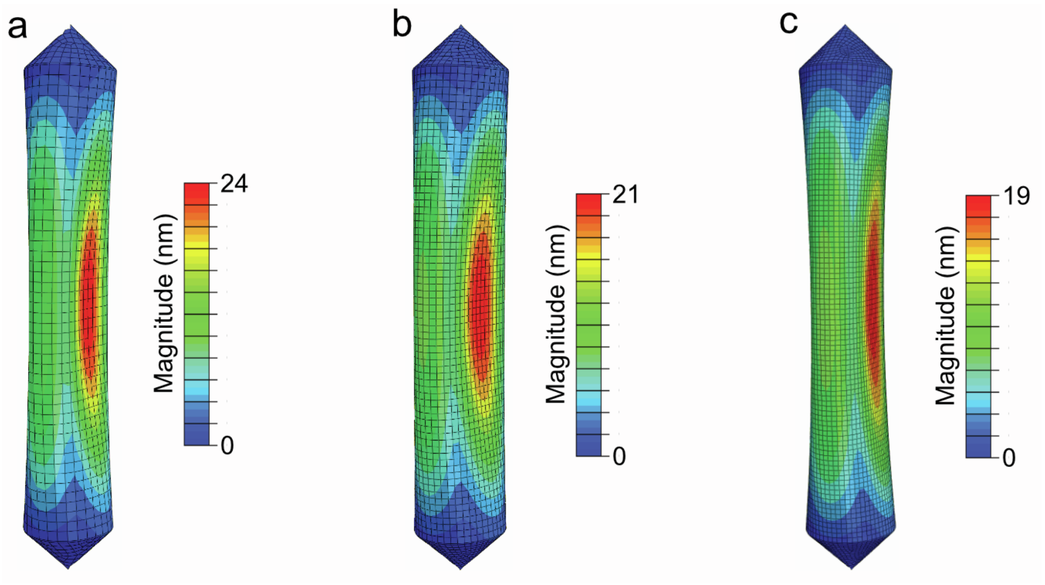

Figure S4. Mesh sensitivity analysis. GV buckling simulations (with GV length and diameter set to 500 nm and 85 nm, respectively) are carried out using three different mesh sizes. A buckled configuration is depicted for each of the three models with discretization lengths as follows: (a) 9 nm, (b) 6 nm, and (c) 4.5 nm. Our results show that all three models predict an identical threshold buckling pressure of 331 kPa.

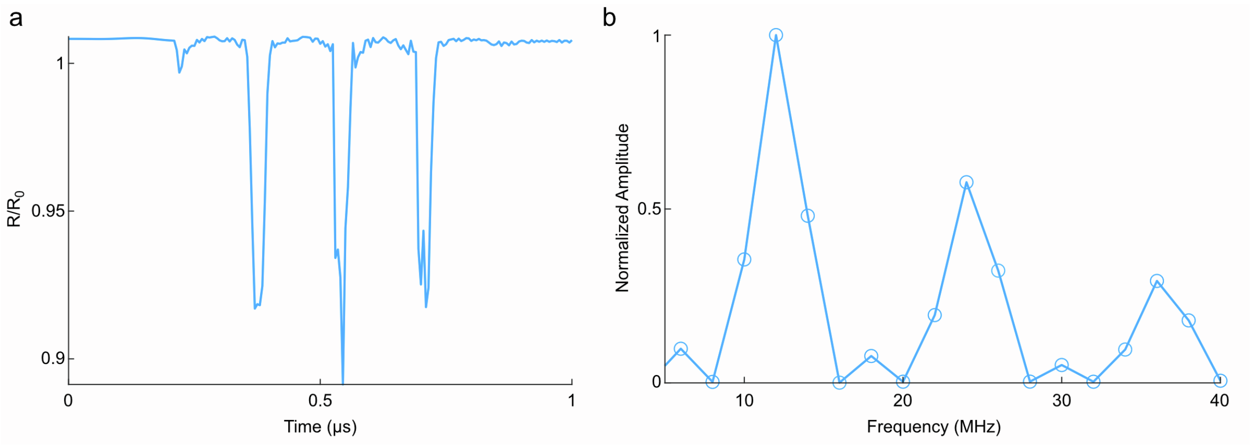

Figure S5. (a) Simulated radius change of AnaS normalized to the original radius (R_0) when exposed to 11.4 MHz and 331 kPa ultrasound. (b) The Fourier transform of GV's radial excursion.

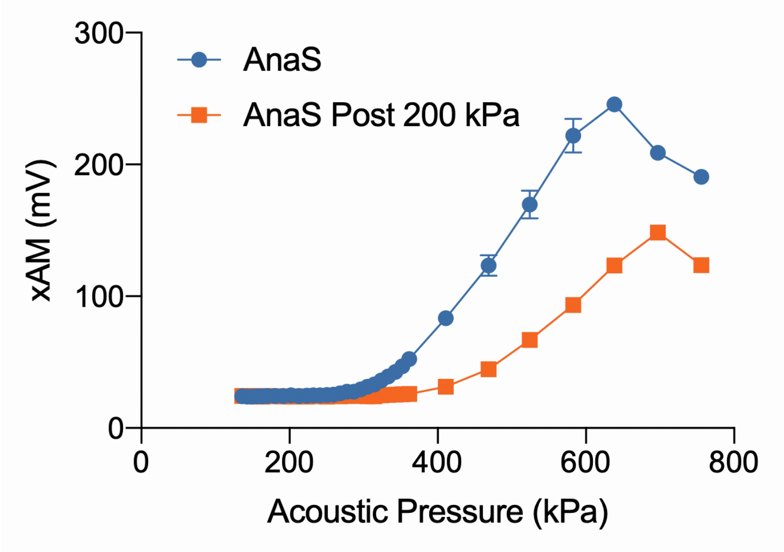

Figure S6. Nonlinear ultrasound signals from AnaS (n = 4) as a function of acoustic pressure from samples with (orange) and without (blue) pre-collapse hydrostatic pressure treatment at 200 kPa. Error bars represent ± SEM, where not seen, are hidden by symbols.

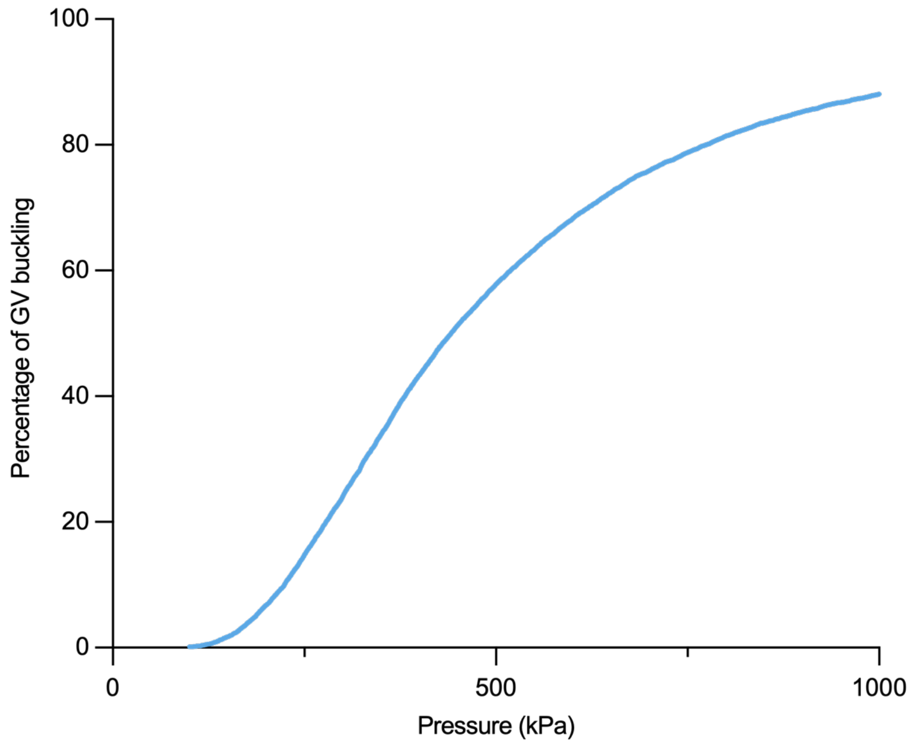

Figure S7. Buckling of an ensemble of 10000 non-interacting GVs with a Gaussian distribution of lengths and diameters, with lengths varying between 500 to 1000 nm and diameters varying between 70 to 100 nm.

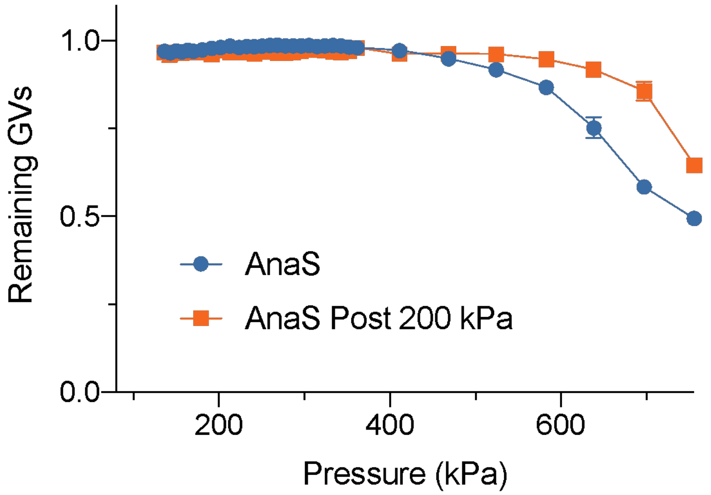

Figure S8. Quantification of remaining GVs by B-mode ultrasound imaging after exposure to different acoustic pressures under xAM imaging. Error bars represent ± SEM, where not seen, are hidden by symbols.
